# LDAF: Low-Bandwidth Distributed Applications Framework in a Use Case of Blockchain-Enabled IoT Devices

**DOI:** 10.3390/s19102337

**Published:** 2019-05-21

**Authors:** Matevž Pustišek, Dejan Dolenc, Andrej Kos

**Affiliations:** Faculty of Electrical Engineering, University of Ljubljana, Tržaska 25, SI-1000 Ljubljana, Slovenia; dejan.dolenc@ltfe.org (D.D.); andrej.kos@fe.uni-lj.si (A.K.)

**Keywords:** fog computing, internet of things, communication constrains, gateway, blockchain

## Abstract

In this paper, we present Low-Bandwidth Distributed Applications Framework (LDAF)—an application-aware gateway for communication-constrained Internet of things (IoT) devices. A modular approach facilitates connecting to existing cloud backend servers and managing message formats and APIs’ native application logic to meet the communication constraints of resource-limited end devices. We investigated options for positioning the LDAF server in fog computing architectures. We demonstrated the approach in three use cases: (i) a simple domain name system (DNS) query from the device to a DNS server, (ii) a complex interaction of a blockchain—based IoT device with a blockchain network, and (iii) difference based patching of binary (system) files at the IoT end devices. In a blockchain smart meter use case we effectively enabled decentralized applications (DApp) for devices that without our solution could not participate in a blockchain network. Employing the more efficient binary content encoding, we reduced the periodic traffic from 16 kB/s to ~1.1 kB/s, i.e., 7% of the initial traffic. With additional optimization of the application protocol in the gateway and message filtering, the periodic traffic was reduced to ~1% of the initial traffic, without any tradeoffs in the application’s functionality or security. Using a function of binary difference we managed to reduce the size of the communication traffic to the end device, at least when the binary patch was smaller than the patching file.

## 1. Introduction

The Internet of things (IoT) has become an enabling infrastructure for a wide range of novel applications. Traditional IoT architectures are cloud-centric [1], where a mash up of cloud services provides the backend for the storage, analysis, visualization, and application of IoT data. This data is collected from several end devices placed in common things or objects, over wireless, mobile, or fixed networks. Emerging approaches in IoT shift the centralized cloud—based architecture towards fog architecture [2], where a part of data storage and processing is moved from the cloud closer to the network’s edge. This reduces application-level latency, scales better to immense amounts of IoT devices, and reduces the core network’s traffic loads. Both cloud- and fog—based approaches rely extensively on application programming interfaces (API) to access data and services at various points within the architecture.

IoT end devices are frequently limited in their computational and storage resources or communication bandwidth, and operate under limited available energy. It is therefore unsurprising that there is a set of platforms, protocols, and messaging formats, as well as networks to support these stringent requirements. They range from efficient embedded IoT platforms [3] over adapted application layer protocols for IoT messaging (e.g., Constrained Application Protocol (CoAP) [4] and Message Queuing Telemetry Transport (MQTT) [5]), to dedicated low power personal areas (e.g., Bluetooth Low Energy (BLE) [6] and IEEE 802.15.4 [7]), low-power long-range networks (e.g., Low Power, Wide Area networking protocol (LoRaWAN) [8] and Narrow-band Internet of Things (NB-IoT) [9]), and sensor and peer-aware (e.g., Wi-Fi Direct [10] and IEEE 802.15.8 [11]) communication networks.

Gateways have long been an essential part of heterogeneous and distributed information, service and communication infrastructures (e.g., signaling and media gateways in VoIP systems, gateway GPRS support node (GGSN) in 2/3G, serving (SGW) and packet data network (PGW) gateways in EPC, etc.). A communication gateway in IoT makes possible the interoperability of heterogeneous communication systems, but can also provide additional communication services, as for example security. An application gateway is finely tuned based on the given application’s requirements. If it is data driven, gateways focus on message and protocol adaptations and optimizations. They can, e.g., provide API harmonization. Application-driven ones assist mediating services [12] and adapt application protocol operations. In the field of IoT, there have been examples of application-specific gateways reported for smart homes, e-health, e-mobility, and including gateways in blockchain—based IoT infrastructures [13]. Gateways may not be independent entities: they can be part of a broader middleware solution. Fog computing led to additional importance for gateways and growth in their functionality. In fog—based IoT solutions, they bring a part of the initial analytics close to the data source and thus facilitate the decentralization of application deployment. Such gateways are no longer just communication and application mediators, but now contribute to the scalability, security, programmability, and real-time features of fog systems [14].

Fog and related mobile edge computing provide architectural approaches that are complementary to traditional cloud-centric systems [15]. Thus computing, storage, networking, and control in modern information and communication systems can be allocated at various points in the system to meet the requirements of emerging applications and challenges imposed by the IoT [14]. These can be stringent latency requirements, network bandwidth constraints, and resource constraints of end devices, intermittent connectivity, or security. We are still searching for the best architectural principles to support IoT by fog [16]. A strong and solid architectural foundation must consider application requirements and be able to balance between these requirements and networking and computing resources. These challenges are additionally pointed out with novel combinations of technologies, such as the IoT supported by the blockchain [17].

Improvements that IoT and blockchain integration can result in decentralization and scalability; device management and authorization; trustworthiness in data; trusted sharing service; data monetization; autonomous machine-to-machine transactions; including smart contract—based decentralized applications; and micropayments. Application opportunities are being sought in areas as smart cities, smart homes, mobility, health, logistic, and food traceability [18]. There are still a great number of open issues that have to be studied in order to seamlessly use IoT and blockchain technologies together. Khan et al. [19] point out several challenges and future research directions for effective implementation of security for IoT devices, including BC. These include resource limitations, device heterogeneity, interoperability of (security) protocols, and scalability and latency of BC networks.

The research in [18] defined three types of BC IoT interactions. In the IoT–IoT approach only a part of IoT data is stored in the BC. End devices predominantly communicate directly (e.g., over a sensor or peer-aware network) without using the BC. This approach has lowest latency. In IoT–BC all interactions go through BC. This ensures that all interactions are traceable, but increases the bandwidth and data in the chain. In hybrid design only a part of the interactions take place in the BC and the rest are directly shared between the IoT devices. The hybrid operation leverages the benefits of BC and low-latency interactions. The fog principle in the hybrid approach can incorporate more powerful devices such as gateways, which can then be used as BC components. This enables the secure participation of constrained devices in blockchain communications. In the same study they presented a resource estimation for various BC clients, including Ethereum, running in RPi v3, but no variations in end device architecture or the impact of communication adaptation were analyzed. Running a light or full blockchain node in a constrained end device is possible, but permanent connectivity beyond low-bandwidth is required. We have various other, more efficient implementation options for the device-to-BC connection, based on remote and trusted BC nodes: (i) we can rely on “of-the-shelf” BC node API. This does not facilitate any application-awareness and adaptation and thus impedes further communication traffic reduction. The second option is (ii) proprietary customization of BC nodes, their APIs, and device communications. Beside the large efforts to maintain such a solution, the legacy approach increases heterogeneity and thus leads to vertical silos, and reduces the interoperability and security of the solutions. BC services can be provided via (iii) cloud APIs, for example, in Hyperledger [20]. This reduces the decentralization of the solution. Our proposed option is (iv) applying application-aware gateways to facilitate BC services for the end devices. Unlike in (iii) the end device can execute BC operations if required (e.g., create transactions, sign transactions, and receive event notifications) or delegate these functions to the gateway. Gateways can be thus fog elements that are located at the edge of the network. A context and application-aware fog smart gateway, and its role in fog computing and cloud of things, is presented in [21], but not specifically for blockchain. In [22] a LoRA implementation of BC aware GW was presented, but no real traffic measurements are given.

Our study was initially motivated by our previous work with blockchain IoT systems. We were focusing on the blockchain IoT client architectures [23] and the challenge of connecting communication constrained IoT devices to the Ethereum blockchain network. To our knowledge, there has been no study of application-aware gateways for IoT and blockchain integration with measurements of real traffic in communication of BC—enabled IoT devices. For the research in this paper we designed an application-aware gateway for constrained devices and implemented its server and client part in NodeJS [24]. We soon realized that such a gateway could support other application examples apart from the blockchain. Its design therefore enables efficient extensions with other application-aware services. We tested the gateway in various application examples. Using it leads to a significant reduction in the required communication traffic to/from the IoT device. It does not aim to reduce latency. Blockchain operation is not real-time and major delays appear there because of the nature of BC transaction validation and not because of the communication or gateway processing delay. For ultralow latency applications, a hybrid approach for BC IoT interactions can be taken [18]. In the blockchain-related use case we effectively enabled decentralized applications (DApp) for the devices that without our solution could not participate in a blockchain network. We adapted the client architecture and applied advanced traffic reduction functions for an Ethereum—based smart-grid switch and counter [25]. Using a binary difference service we managed to reduce the size of the communication traffic to the end device as long as the binary patch was smaller than the patching file.

Beside the evident impact in reduction of communication traffic, the proposed solution maintains high flexibility and can be easily adapted to, e.g., changes in remote APIs, or can accommodate other/new communication and application protocols.

In our research we performed the following.

- Designed and implemented an application-aware gateway, LDAF, which can be a part of a fog computing architecture. It enables fog data services for reduction of communication traffic of bandwidth constrained IoT devices to connect them to arbitrary cloud APIs.
- We set-up and executed a decentralized application use case with blockchain-enabled IoT devices, where the LDAF gateway was applied and adapted to the application’s operation, to reduce the size and number of messages needed for the IoT application.
- For blockchain-enabled IoT devices we measured that the binary encoding of message content reduced the periodic traffic from 16 kB/s to ~1.1 kB/s, i.e., 7% of the initial traffic. Additional optimization of the application protocol in the gateway and message filtering further reduced the periodic traffic to ~1% of the initial traffic, without any tradeoffs in the application’s functionality or security. In two other supporting use cases we demonstrated the possible applicability of LDAF for other fog services.

In Section 2 we briefly present the adaptations of the communication networks and protocol stacks in IoT systems. Section 3 elaborates the architecture and objectives of the LDAF framework and Section 4 positions it in the modern fog architecture. In Section 5 we present three use cases where LDAF was applied to optimize communication traffic of constrained IoT devices. Section 6 concludes our research and highlights further research and application options.

## 2. Background

IoT device ecosystems impose changed and shifted priorities for end devices. IoT devices often require long battery life, low deployment and maintenance costs, long operation lifetime, and careful conservation of devices and network resources [26]. These affect the communication networks and protocol stacks in IoT systems. For example, new low-power short (LP-PAN) and long-range (LP-WAN) technologies were developed. Networks like Sigfox, LoRaWAN [8], or NB-IoT [9] are especially dedicated to low bitrate and low power long-range connectivity, which was not achievable in traditional mobile or wireless networks. New radio transmission schemes and physical channels are applied. IoT devices can remain in sleep mode for the majority of their lifetimes to prolong energy autonomy. These networks are being deployed around the globe and readily provide free or commercial IoT communication services. Device connectivity can be provided with short-range technologies, too. In this case devices connect to a gateway that has a persistent internet connection. LP-PAN includes, e.g., Bluetooth (BT) [6,27] or IEEE 802.15.4 [7]. The latter specifies the physical layer and media access control for low data-rate, low-power, and low-complexity, short-range radio frequency transmissions in wireless personal area networks. It can be further complemented by 6LoWPAN [28], which defines a binding for the IPv6 WPANs. Additional advancements in wireless networking can be found in peer-aware communications (PAC). IEEE 802.15.8 PAC [11] is WPAN technology optimized for peer-to-peer and infrastructure-less communications with fully distributed coordination. IEEE 802.15.8 PAC features include discovery for peer information without association, a discovery signaling rate typically greater than 100 kb/s, detection of the number of devices in the network, and scalable data transmission rates. Wi-Fi Peer-to-Peer v1.7 [10] is the specification for the Wi-Fi Alliance Wi-Fi CERTIFIED Wi-Fi Direct^®^ program, which allows Wi-Fi client devices to connect directly with each other by setting up ad-hoc networks, without going through a wireless access point or hotspot. In hybrid IoT–BC systems, PAC and Wi-Fi Direct can complement the IoT–BC interactions where low-latency or lager peer communication bandwidth is required.

The IoT protocol stack demonstrates new or adapted transport and application protocols. The TCP/IP header and protocol redundancy can become an issue if low-bandwidth communications are sought. At the transport layer the simpler UDP can be applied instead of TCP. However, as it only provides a best-effort transport service, an adapted security protocol DTLS was needed to operate over UDP. DTLS [29] implements its own sequencing and simple packet retransmission, which would otherwise be a part of the TCP. In an extreme case, IoT stack can renounce the TCP and IP and apply optimized non-IP networking and transport solutions, as for example the non-IP data delivery (NIDD) [30] over NB-IoT (a part of 3GPP Release 13 Features) or in device-to-gateway communication through LoRaWAN MAC.

The application layers Constrained Application Protocol (CoAP) [4] and Message Queuing Telemetry Transport (MQTT) [5] provide low(er) bitrate alternatives to the established HTTP(S) [31,32] and WebSockets [33]. CoAP is a one-to-one request-response protocol for transferring state information between client and server. It is a simpler alternative to HTTP, which results in simpler hardware requirements for CoAP smart objects, as well as in the protocol’s lower communication overhead and therefore of the resulting reduced power consumption [34]. MQTT is a publish-subscribe protocol: a broker decides where to copy and route messages published by the clients. This enables a many-to-many bus for live data communication. Clients mostly apply a TCP connection to the broker. MQTT is not optimized for low-bitrate communications; however, the MQTT for Sensor Networks (MQTT-SN) extends the MQTT for low-power and low-cost devices. It does not require TCP/IP stack, and can be alternatively used over UDP.

Apart from the aforementioned CoAP and MQTT, additional application-layer protocols strengthen support for IoT devices [35]. The OMA LWM2M protocol [26], for example, introduces device management functionality and transfer of service data from the network to devices over sensor or cellular networks. It is based on CoAP and DTLS, but other connection bindings are supported, too.

Despite various adaptations of communication networks and protocol stacks for IoT, cloud-centric IoT systems apply more common communication, transport, and application technologies to make the cloud resources available to others. These cloud systems range from, e.g., IoT collection and analysis platforms [36], public open-data resources [37], geolocation and maps [38], open weather information [39], and similar sources. The technologies for cloud APIs are based on TCP/IP stack with HTTP or WebSocket at the application layer. If structured data formats are applied for content upload/download, these are commonly JSON or XML. We have three options for matching IoT device-specific communications and message formats with those of the cloud backend:

(i) Support IoT specific protocols in cloud APIs: not likely, if the cloud service is externally provided and the IoT solution developer therefore cannot affect the cloud API implementation and functionality.
(ii) Develop a dedicated proxy function between the available API implementation and the one appropriate for our IoT devices.
(iii) Apply a modular application gateway, as the LDAF presented in this paper, where common API and device connectivity functions are readily available and where the developers can concentrate on application-specific optimization of traffic volumes.

## 3. LDAF Background and Overview

**Figure 1:**
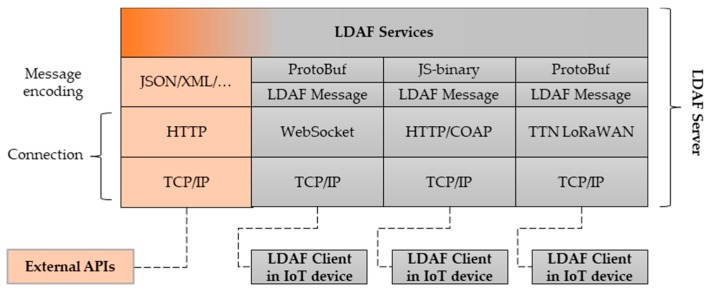
Overall architecture of low-bandwidth distributed application framework (LDAF): LDAF Server, LDAF Clients, and external application programming interfaces (APIs).

The Low-bandwidth Distributed Application Framework (LDAF) [40] is comprised of a LDAF server and corresponding client entities—Figure 1. A LDAF client runs on an end device, e.g., an (constrained) IoT device, and connects to the LDAF server via one of the available connection mechanisms. At the moment WebSockets, HTTP, and The Things Network (TTN) [41] are implemented. The LDAF server connects to arbitrary cloud APIs, e.g., cloud platforms for IoT, Google services, Ethereum client API, etc., to enable the following.

- API aggregation: the LDAF server adapts to specific external API requirements (communication protocols, message formats, and authentication) and adapts it to a common message exchange between itself and the client.
- Efficient binary message serialization: the content of the messages between the client and the server is binary encoded using Protocol buffers (ProtoBuf, PB) [42] or similar binary encoding mechanism to reduce the message size. This is especially efficient compared to exchanging structured text—based messages (e.g., JSON and XML).
- Application adaptation: server can adapt the application logic, e.g., to reduce the number of messages passed to the client to further reduce the communication requirements. It can also take over a part of a device’s application execution (heavy computations, hash calculations, caching, etc.), and thus not only reduces the communication bitrate, but the processing and storage requirements at the end device too. This allows for applications beyond simple aggregation of APIs.

The LDAF framework is characterized by modular architecture, as depicted in Figure 1. It enables the flexible addition of new connection mechanisms and new services. A service is a particular mapping between an API, or APIs, and the clients that are communicating with these APIs through the LDAF server. There can be an arbitrary number of services implemented in LDAF server.

Without LDAF the device would connect directly to the external API, and would therefore have to comply with the protocols, syntax, semantics, and application logic imposed by the API. These might not be well adapted to the device constraints.

### 3.1. LDAF Architecture

#### 3.1.1. Messages

**Figure 2:**
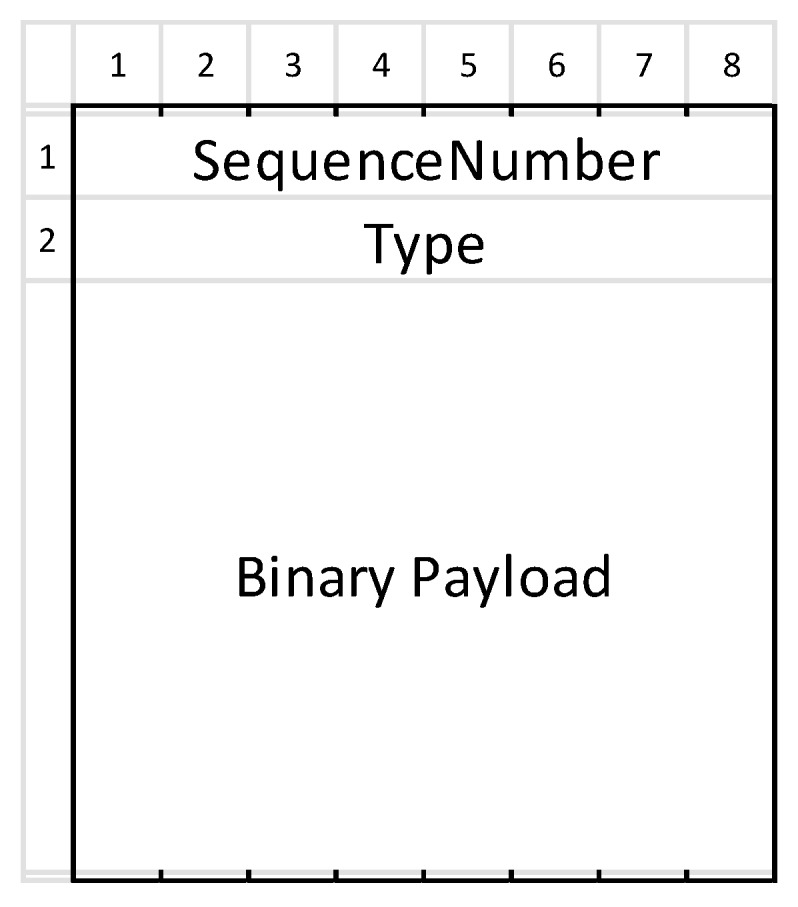
LDAF message format.

A LDAF message is comprised of a header and payload. As can be seen in Figure 2, the header has two fields, namely Type and SequenceNumber. With default settings each field is 1 B long, but can be reconfigured if more message types and longer sequences are required.

A message sequence is required to associate the responses with the appropriate request. However, it could be applied in the future to increase the reliability of message transfer at the application layer (reordering message sequences, detecting message loss, ARQ). In push messages from the server to the client the SequenceNumber is omitted because such a message is not a response to a specific request.

The message type determines which schema must be applied to decode the binary message content. Both the message Type and the SequenceNumber are emitted to the available Services, so that the corresponding Service decodes and processes the message.

There can be an arbitrary number of message types required by one Service. This would make static type assignments inefficient; we therefore applied a dynamic assignment. The message type is indicated by the value at the corresponding location in the Type parameter. The Type parameter is interpreted by a service offset, which points to the first message type for each particular service. The following service offset points to the location after the last message type of the previous service. The offsets of all services applied in a connection are specified in the service definition.

We thus minimized the data required for the appropriate type identification. For each message we thus introduced 2 B of additional overhead and only 1 B for push messages. The remainder is the payload. With 1 B for the Type field we can organize, e.g., 1 Service with up to 256 different messages types or two Services, one with 20 and the other with up to 236 types. For the Ethereum—based smart grid meter, for example, four types of messages are required: (i) a periodic block notification, (ii) an event notification, (iii) a request, and (iv) a response for transaction verification.

LDAF messages are thus encoded in two substages. The first is the efficient binary serialization of the message content (e.g., protocol buffers [42] and js-binary), and the second is building the message header, which consists of SequenceNumber and Type, as depicted in Figure 2 and explained previously. If required, other serialization methods can be included in the framework.

#### 3.1.2. Connections

A LDAF connection determines how end devices connect to the LDAF server. There can be various options implemented to meet the needs of the constrained devices. Current implementation supports WebSockets (over TCP/IP), HTTP (over TCP/IP) and integration with the LoRaWAN and The Things Network [41]. In a similar way, CoAP (over UDP/IP) or MQTT can be added.

A connection receives and forwards messages from a device to the corresponding Service. During the connection setup, service definitions at the LDAF client and at the LDAF server are compared. If they do not match (e.g., client requesting unavailable services), the connection is not established. This assures that the server can provide all the required services, that the message types are correctly interpreted, and that the message payload is correctly decoded.

#### 3.1.3. Services

Services address the needs of end device applications and therefore affect the size and/or number of messages for the end device. Two levels of application adaption are possible:

- Transcoding of messages: The service receives messages from the external APIs in format and protocol imposed by the API and transcodes the payload to the LDAF message format described in Section 3.1.1. In this case the application does not affect the number of messages from/to the device.
- Application adaptation: The service in this case actively modifies the application logic. This affects the number of messages and further reduces the traffic for the end device. The service can determine whether a particular message is actually required for the device application and filter it out. It can, e.g., aggregate duplicated messages, negotiate and confirm message exchange with the API, and only pass the success notification to the end device, or build an aggregated response for the device that includes data collected from various APIs.

Application adaptation helps LDAF servers distribute the application logic among the end device, cloud services, and the edge of the network.

#### 3.1.4. Server and Client

The LDAF framework is comprised of server and client entities. The LDAF server embodies the establishment and management of the end device connections, message encoding, service execution, and connections to external APIs. The LDAF client applies a set of client libraries for the LDAF server connections and message encoding and enables end device application logic to communicate with the framework.

When the LDAF server is initialized, the Service.js library scans the subdirectory where all available services are defined and implemented. A particular service is usually implemented as a main library (main.js), with the specific application logic and a service definition file (serviceDef.js). Service definition files specify message type object. It also specifies (binary) message-content encoding mechanism, including corresponding message definition files (e.g., proto). Upon initialization, connections to the external APIs are established, too.

**Figure 3:**
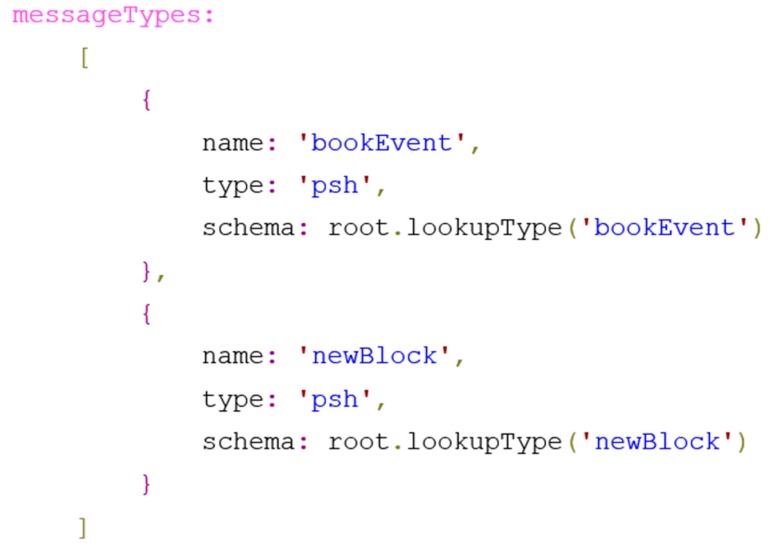
Message type definition for the Ethereum—based smart grid switch and counter service.

We applied two message types for the Ethereum—based smart-grid switch and counter. As seen in Figure 3, one is for notifications about new blocks added to the blockchain (‘newBlock’) and the other (‘newEvent’) for new event notifications sent from the smart contract to the device.

When a new client connects to a selected service on an LDAF server, they compare client and server service definitions, which must match to ensure correct encoding and decoding of messages. Both service definition files (serviceDef.js) and message definition files (e.g., proto) must be present on the client’s side, too. We provide a generic client library (Client.js), which establishes the connection to the server and receives, transcodes, and transmits messages.

## 4. Positioning the LDAF in Fog Architectures

There are several options for deployment of LDAF server in current and future communication networks. These options differ in their level of network integration. They can range from

(i) Function reachable through the network and implemented at (external) application servers, which are only connected, but not really integrated in the network infrastructure, and
(ii) Function provided by the network, where LDAF is a functional component of, e.g., the 4G/5G network.

Applying LDAF as an additional application server reachable through the network is a straightforward approach. It is independent from the underlying network infrastructure. The network is merely providing internet connectivity from end devices to the LDAF proxy. In LoRaWAN the application servers are already foreseen in the architecture. We tested this approach with a LoRaWAN TTN platform. In a similar manner the current 4G and emerging 5G networks provide channels to external internet servers and applications. This approach however, cannot fully exploit the possibilities that would appeared if LDAF were integrated into the network infrastructure.

The benefits of integrating LDAF in the network infrastructure—and thus the gateway being a function provided by the network—may include the following.

(i) Proximity of computational and storage resources to reduce latency and backend traffic.
(ii) Collaborative sensing where LDAF would implement initial data preprocessing and aggregation of messages or streams from several end devices.
(iii) Switch to non-IP data delivery for the end device for further traffic optimization.
(iv) Information about the status of the radio-access network (RAN) for dynamic LDAF services or near-real-time application-aware performance optimization of the LDAF services.
(v) Context awareness of the services, where context can reach beyond traditional cookie or location—based adaptations.

LoRaWAN network architecture is typically laid out in a star-of-stars topology, in which the gateway is a transparent bridge relaying messages between end devices and a central network server in the backend. Gateways are connected to the network server (NS) via standard IP connections, while end devices use single-hop wireless communication to one or many gateways. The network’s server hosts all the intelligence; it manages packets from different gateways and sends them to the specific application server. In LoRaWAN, LDAF could become a part of the network server. A similar approach was taken in [43], where the NS blockchain management of end devices was built into LoRaWAN architecture. LDAF services in NS could rely on the network and device status provided by the NS and thus adapt service operation. The LDAF could, e.g., pass on the time-insensitive information only when the end device wakes from sleep mode for other communications, without additionally awakening it.

In mobile edge computing (MEC) [44], Evolved NodeB (eNB) and Next-generation NodeB (gNB) not only provide radio access, but can host the fog nodes that execute a part of the application’s functionality at the edge of the network. A new feature of LTE and 5G networks, as compared to 3G, is the exchange of data flows directly among the neighboring NBs. These would enable new use cases and applications like computation offloading, distributed content delivery, web performance enhancements, and application-awareness and optimization for IoT [45]. The LDAF server placed at the NBs could, e.g., aggregate and exchange data from various end devices, including those connected to different radio nodes. Besides, the network slicing mechanism [46] completely separates the network service provision from the actual physical infrastructure. The network, storage, and computational resources can be dynamically allocated in a network slice. In 5G networks it is already foreseen that the computational and storage capabilities for the control and data plane may be positioned at various locations in the network topology.

The LDAF proxy could be integrated in wired power-line communication systems (PLC), which are important customer–domain infrastructure in smart grid systems. The predominant use of PLCat the moment is remote meter reading; however, new smart grid applications envisage new communication over PLC. A PLC modem is integrated in the (smart) electricity meter on the customer’s side. The PLC communication is terminated in a message-aggregating gateway, located at the secondary substation. A secondary substation usually has its own local area network and wired broadband connectivity towards the higher levels of the grid’s architecture. The PLC in such a setup only provides low data rates, which limits the frequency of meter reading and practically narrows the scope of new associated services. An LDAF server would be placed at the message-aggregating gateway and the LDAF client in smart meters.

## 5. Use Cases

To demonstrate the applicability of our approach we set up three use cases and applied the LDAF framework to manage the traffic from/to a communication-constrained device. The first case is a simple DNS query in which a device resolves a given host name to a corresponding IP address.

The second example represents a realistic case of an IoT device, controlled over the Ethereum network. For this we applied our previously developed prototype system, dubbed Swether [25]. This is an IoT electric meter and switch, which is a part of a broader decentralized application (DApp). The DApp is comprised also of a corresponding smart contract deployed in the Ethereum network and a web—based Ethereum enabled user interfaces to initiate control activities. The application logic in Swether requires access to a fully synchronized Ethereum blockchain (BC) node. In our case the node was set on a remote server. Without LDAF the Swether connects to the BC node’s JSON-RPC API over WebSockets. The remote server provides it with key BC functionalities, including indication of chain synchronization, event notifications from the smart contract, and creation, signing, and deployment of Ethereum transactions. The details of this architecture are discussed in [23].

The third case targets binary transfers from the network to the IoT end devices, as, for example, in software updates and patching. To reduce unnecessary traffic to the device, we implemented a service that, instead of forwarding a complete new binary file to a device, calculates and forwards only the binary difference between the old and new version of the file. As in other cases, the originating file server does not implement such a service and only provides full versions of files. We can apply such a service with Swether, too, e.g., for system updates.

### 5.1. Measurement Setup

**Figure 4:**
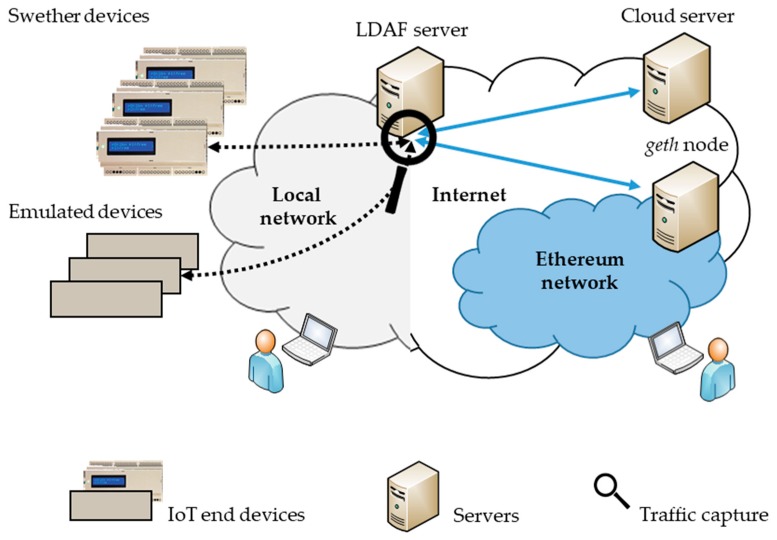
Measurement setup.

To execute and evaluate the described use cases, we set up the environment depicted in Figure 4. One Linux based computer hosted the LDAF server. It was connected to the Internet to access the APIs of external (cloud) applications (e.g., the Ethereum geth client for BC or any cloud API according to the application needs). It was also connected to a local IP network for communication with IoT end devices. If necessary the local IP network could be replaced by non-IP access, e.g., by integrating the LDAF server into LoRaWAN access point or network server.

In the blockchain use case, the end devices were five physical instances of the Swether device, as well as several emulated instances. For emulation only the specific sensing and actuation hardware was eliminated, otherwise they ran exactly the same application software as the Swether, and thus producing the same network load. The emulated instances were applied to create a number of concurrent clients while estimating scalability of the system. Beside the LDAF server, in the BC use case another server was applied, to host the Ethereum geth client. It had permanent Internet connection to keep the geth client synchronized with public the Ethereum network. For DNS and binary transfers to the IoT devices, corresponding HTTP request were launched from the end devices to the LDAF server, when needed.

The entire communication traffic from/to LDAF server was captured and analyzed with Wireshark.

#### Resource Consumptions at the LDAF Server

During the operation we estimated the system requirements imposed to the LDAF server. Because the CPU and memory requirements were so low, we installed the LDAF server on a Raspberry Pi (RPi) 3 B as well. We tested it with up to 100 concurrent clients using the blockchain service in LDAF. The memory consumption was independent from the number of clients and was ~5%, i.e., 50 MB. The CPU load was varying. During the block processing (the Ethereum network creates a new block approximately every 20 s) the 1 s average was approximately 3–6%, otherwise ~1%. LDAF service implementation assures that the number of clients subscribed to the same service, does not significantly affect the CPU load. We would like to point out that a systematic resource monitoring of the server resources was not our research objective. The observed values can serve as indication and are application-specific and indication and are application-specific.

### 5.2. Example 1: DNS

We developed a new service definition (dnsServiceDef.js) and a simple DNS client. The LDAF client initiates a WebSocket connection for the transaction between the client and the LDAF server. The LDAF server sends a DNS query to the predefined DNS server and receives the responses. These transactions are encapsulated in the UDP messages. The service definition includes the parsing rules from the DNS response of the DNS server and forms message syntax for the LDAF client.

**Figure 5:**
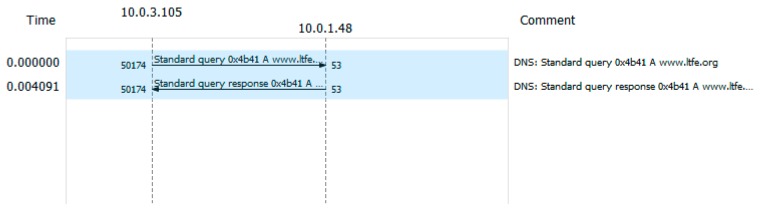
Regular DNS transaction from NodeJS environment.

The regular DNS transaction executed by the NodeJS environment results in one request and one response, as shown in Figure 5. The sizes of the messages are 72 B and 88 B, respectively. The DNS data sizes are 30 B and 46 B. The request size might depend on the particular domain name.

**Figure 6:**
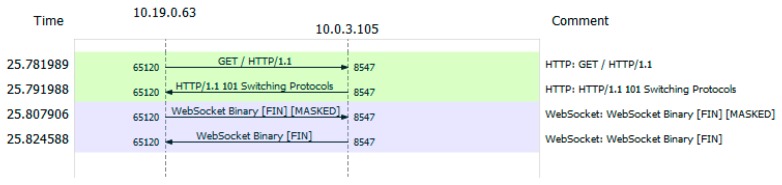
Transaction between the LDAF client and the server.

Figure 6 presents the corresponding message sequence between the LDAF client and LDAF server. An HTTP request and response are required to set up the WebSocket. Once the socket is up, there is one request and one response in the transaction. The sizes of the messages are 75 B and 72 B, respectively. The WS data is 15 B and 16 B. Although the LDAF does not address low-latency requirements for IoT applications, we estimated the delay introduced by the application of LDAF server and service. For DNS without LDAF the response time was about 4 ms. The LDAF added ~40 ms including the additional delay for TCP session setup.

The sizes of the messages in regular DNS transactions and in LDAF are comparable. This is because DNS is not a communication-intensive protocol. It applies UDP for transport and therefore gains an advantage of 12 B per packet over WebSocket using TCP. However, if we focus only on data fields in both transactions, the difference is more evident. Binary encoding in LDAF reduces the size of the message content field of all messages to about 1/5.

### 5.3. Example 2: Ethereum—Based Smart Grid Switch and Counter

We developed a new service definition (serviceDef.js) for LDAF server and the client was a Swether device configured to operate over LDAF. Upon service initiation the LDAF server connects to the remote Ethereum node. Just as in case of DNS, also here the client initiates a WebSocket connection for communication between the client and the LDAF server. The LDAF in our case enables two optimization scenarios:

1. Binary message content serialization by applying binary encoding of message content instead of textual (JSON). In this case all the messages from the Ethereum node are passed to the Swether. Bandwidth reduction is a consequence of more efficient encoding.
2. Application adaptation reduces the number of block notifications that are periodically sent from the Ethereum node to the connected devices. For Swether, we applied this information to check the block depth for security reasons. After receiving an event notification for an action to be executed in the Swether, it first waits for a predefined number of blocks to verify that the notification is actually placed deep enough in the chain. However, in most other cases the block number notification is not required by the end device.

We therefore implemented block notification filtering at the LDAF (with an arbitrary filtering ratio), to reduce the number of messages. To maintain the application’s security, event validation was implemented in the LDAF service, too. Only verified event notifications are passed to the Swether. As Swether does not have to verify them, block number notification is no longer essential data for application operation. Only occasional block number notifications are applied to check the node synchronization.

For the first scenario we set the LDAF service to pass every (1 out of 1) block notification to the Swether. We captured all the traffic from and to the LDAF server, so as to include the WebSocket session setup and at least 10 consecutive block notifications. The traffic between LDAF server and Swether was 1104 B and between the LDAF server and the Ethereum node about 16 KB. Due to binary message content, encoding the traffic was thus reduced to ~7% of that from the original API of the Ethereum node. For DNS, this advantage was minimal due to the small DNS query sizes and UDP. For Ethereum the reduction is substantial, as the traffic is reduced nearly to 1/14.

**Figure 7:**
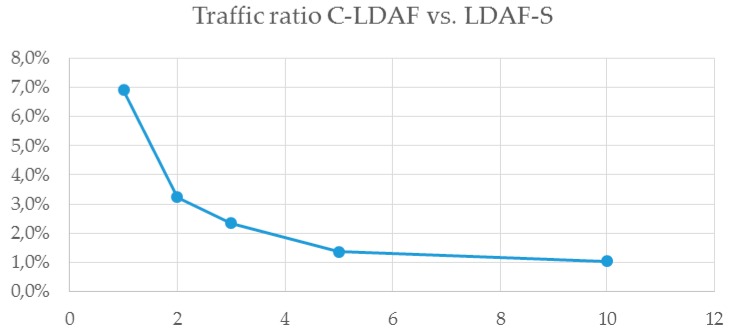
Traffic ratio with packet filtering: communication between the LDAF client and LDAF server vs. LDAF server and the Ethereum node.

However, the traffic was even more reduced when the application adaptation was applied. As shown in Figure 7, we applied different filtering rates to reduce the number of block notifications for the Swether. If we reduced the number to one out of two messages, the traffic was ~3% of the initial sum. If we let every one out of 10 messages through, the remaining traffic was only 1%. Despite the evident traffic reduction, we did not make any tradeoffs in the application’s functionality or security.

### 5.4. Example 3: Binary Transfers to the End Device

We developed a new service definition (serviceDef.js) for LDAF based on the bsdiff-nodejs library [47] to enable the following.

Application adaptation. The LDAF server caches a version of a previously downloaded file. Upon request from the device or a push from the cloud server for an update, the LDAF service calculates the binary difference between the new and previous content and returns it to the device. The device patches the old version of the file.

Despite binary content, binary LDAF message content serialization still takes place. It is a generic part of LDAF message encoding and not a part of the service. However, it does not additionally reduce the size of the already compressed binary content. If LDAF was applied exclusively for transferring binary files, the binary difference could be implemented as the default LDAF message content serialization. However, being a service allows for the broader use of the framework.

The effect of this service predominantly depends on the binary difference of the two files transferred. To test the operation and estimate the impact of message and communication overhead, we used two binary installation files, named v62 and v63, each ~1.8 MB. The size of the binary difference patch to upgrade from v62 to v63 (as reported by bsdiff) was 1531 kB. The size of the patch when the v62 file was compared to itself was 145 B, to allow for the mandatory bsdiff patch header. Transferring the v63 file from the file server to the LDAF server resulted in 1942 kB in download. The corresponding patch from the LDAF server to the end device resulted in 1627 kB download. The difference between the original file v63 and the patch was 292 kB. The difference in download traffic was even a bit bigger, 315 kB, as a consequence of more efficient WS download compared to HTTPS. The void patch for v62 to v62 resulted in only 619 B download, and that was mostly due to WebSocket establishment. The exact communication traffic loads might vary a little bit, if the experiment was repeated, due to changed network conditions (e.g., more or less frequent retransmissions of lost packets).

Other file pairs would result in larger or smaller binary differences, but the key is that the LDAF framework reduces the size of the communication traffic at least as much as the binary patch is smaller than the patching file.

## 6. Conclusions

We designed, developed, and tested an application-aware gateway framework for constrained devices—the LDAF. It facilitates flexible implementation proxying services to reduce the communication loads by efficiently encoding binary message content and adapting application protocol operation to affect the number of exchanged messages; it further reduces the traffic for the end device. The implementation proved to be robust, able to run several concurrent services and serving various clients.

Currently we are implementing LDAF into a smart city ecosystem (EkoSMART). A part of this project is an integration platform, which supports a variety of smart city applications, including e-health, well-being, and smart mobility. These subsystems contribute IoT measurements into the integration platform and share it to various cross-domain applications. LDAF will enable the connection of communication-constrained devices and API integration.

Our further research is focused on implementing an LDAF server on a LoRaWAN network server for application services that are aware of the network status.

The next foreseen use cases are university e-learning and study management platforms. These platforms are independent, yet they must be synchronized. The available REST APIs, however, are not flexible enough to reduce the amount of traffic required to synchronize the two. This is not an IoT case and the traffic volumes are expected to be substantially higher. At the moment, ~15 MB of data is transferred for periodic syncing, although only ~1 MB is actually required. With LDAF we expect to lower the communication and processing during one synchronization, and thus enable more frequent synchronizations.
